# All but Small: miRNAs from Wharton’s Jelly-Mesenchymal Stromal Cell Small Extracellular Vesicles Rescue Premature White Matter Injury after Intranasal Administration

**DOI:** 10.3390/cells13060543

**Published:** 2024-03-19

**Authors:** Vera Tscherrig, Marel Steinfort, Valérie Haesler, Daniel Surbek, Andreina Schoeberlein, Marianne Simone Joerger-Messerli

**Affiliations:** 1 Department of Obstetrics and Feto-maternal Medicine, Inselspital, Bern University Hospital, University of Bern, 3010 Bern, Switzerland; marel.steinfort@unibe.ch (M.S.);; 2 Department for BioMedical Research (DBMR), University of Bern, 3008 Bern, Switzerland; 3 Graduate School for Cellular and Biomedical Sciences (GCB), University of Bern, 3012 Bern, Switzerland

**Keywords:** premature white matter injury, Wharton’s jelly mesenchymal stromal cells, small extracellular vesicles, in vivo biodistribution, microRNAs

## Abstract

White matter injury (WMI) is a common neurological issue in premature-born neonates, often causing long-term disabilities. We recently demonstrated a key beneficial role of Wharton’s jelly mesenchymal stromal cell-derived small extracellular vesicles (WJ-MSC-sEVs) microRNAs (miRNAs) in WMI-related processes in vitro. Here, we studied the functions of WJ-MSC-sEV miRNAs in vivo using a preclinical rat model of premature WMI. Premature WMI was induced in rat pups through inflammation and hypoxia-ischemia. Small EVs were purified from the culture supernatant of human WJ-MSCs. The capacity of WJ-MSC-sEV-derived miRNAs to decrease microglia activation and promote oligodendrocyte maturation was evaluated by knocking down (k.d) *DROSHA* in WJ-MSCs, releasing sEVs containing significantly less mature miRNAs. Wharton’s jelly MSC-sEVs intranasally administrated 24 h upon injury reached the brain within 1 h, remained detectable for at least 24 h, significantly reduced microglial activation, and promoted oligodendrocyte maturation. The *DROSHA* k.d in WJ-MSCs lowered the therapeutic capabilities of sEVs in experimental premature WMI. Our results strongly indicate the relevance of miRNAs in the therapeutic abilities of WJ-MSC-sEVs in premature WMI in vivo, opening the path to clinical application.

## 1. Introduction

Approximately 10% of all neonates worldwide are born prematurely, defined as birth before the completed 37th week of gestation. Every year, premature birth (PMB) results in the death of one million children before the age of five, making it the primary cause of child mortality [1]. The most common neurological complication underlying PMB is white matter injury (WMI) [2]. In recent years, the incidence of the more severe subtype of WMI, cystic periventricular leukomalacia (cPVL), has decreased. Nevertheless, the more subtle type, diffuse WMI, remains persistent [3,4]. Diffuse WMI leads to long-term neurodevelopmental and neurobehavioral impairments, resulting in severe cognitive and motor deficits until early adulthood [5,6,7,8]. The intellectual performance and overall well-being of WMI survivors are limited, leading to high economic and health burdens [1,9].

Inflammatory and hypoxic-ischemic insults are the main contributors to premature WMI [2,3,10]. They induce an increase in reactive oxygen species (ROS), leading to the activation of microglia and subsequent release of pro-inflammatory cytokines [2,10]. The critical time window for premature neonates to develop WMI is around 28 weeks of gestation. During this time, the developing oligodendrocytes (OLs) are particularly susceptible to harmful insults [11,12]. Activated microglia mainly interfere with the differentiation of the OL lineage, leading to an arrest of OL maturation. The cells remain at the stage of OL-precursor cells (OPCs), which are unable to produce the insulating myelin sheaths of the axons. The hampered myelination impairs neurodevelopment, resulting in adverse motor and cognitive sequelae [12,13,14,15].

In addition to the damage of the white matter, several neuropathological studies of premature-born neonates have revealed apoptosis in grey matter structures after premature WMI [16,17,18]. These structures include the hippocampus, the thalamus, subplate neurons, and the cortex.

Currently, there is no definitive treatment strategy for WMI, and existing approaches have shown limited efficacy, highlighting the urgent need to find a more effective therapeutic intervention to improve WMI outcomes.

Several research groups have found that small extracellular vesicles (sEVs) from mesenchymal stromal cells (MSC-sEVs) offer a promising therapeutic agent for neurological disorders [19,20,21,22,23]. We and others have shown the neuroprotective and therapeutic abilities of MSC-sEVs in premature hypoxic-ischemic brain injuries [24,25,26,27,28,29,30]. We could demonstrate that MSC-sEVs derived from Wharton’s jelly of human umbilical cord tissue (WJ-MSC-sEVs) interfered with key processes involved in premature birth-related WMI. Thereby, WJ-MSC-sEVs reduced microglia-mediated neuroinflammation by inhibiting NF-кB and MAPK signaling pathways [29], prevented and resolved hypoxia-ischemia-induced apoptosis in neuronal cells [30,31], and triggered oligodendrocyte differentiation by restraining Notch and MAPK signaling cascades [32]. Furthermore, we have found that WJ-MSC-sEVs reduced mortality and improved learning and memory, assessed through the use of the Morris water maze assay in an experimental rat model of premature birth-related WMI [30].

Small EVs offer several advantages over cell-based therapies, including lower toxicity and immunogenic properties and the capability to cross biological barriers [33]. The sEVs contain a lipid bilayer, protecting the cargo from degradation [34]. The cargo of sEVs comprises several biologically active agents, including proteins, DNA, RNA, and microRNAs (miRNAs) [35].

In the past couple of years, the interest in the miRNA cargo of sEVs and their effect after the release by sEVs into target cells has been emerging. As we showed that WJ-MSC-sEVs released their RNA content into target cells, miRNAs as key functional units in the neuroprotective and therapeutic effects of WJ-MSC-sEVs moved to the center of our research interest [31]. MicroRNAs are small, non-coding RNAs that range in size between 19 and 25 nucleotides. The processing of miRNAs begins in the nucleus, where they are transformed from primary (pri)-miRNAs to precursor (pre)-miRNAs by a complex involving Drosha, an RNase III endonuclease, and DiGeorge syndrome critical region 8 (DGCR8) [36]. Exportin-5 and the Drosha-DGCR8 complex mediate the export of the pre-miRNAs from the nucleus into the cytoplasm [37]. In the cytoplasm, pre-miRNAs are further processed into mature miRNAs, which are able to bind to the RNA-induced silencing complex (RISC). The miRNA–RISC complex can interact with the complementary 3′-untranslated regions (3′UTR) of target mRNAs, inducing mRNA degradation or translational repression [36]. Mature miRNAs are crucial for gene regulation and, therefore, essential for many physiological and pathological processes [36]. A reduction in *DROSHA* expression results in a significant decrease in mature miRNAs [38]. We have recently shown that introducing a *DROSHA* k.d is a validated method to efficiently assess the impact of miRNAs from WJ-MSC-sEVs in different assays, as it resulted in a decreased amount of mature miRNAs in WJ-MSC-sEVs without altering the physical characteristics of the isolated sEVs [39]. This is in line with the study conducted by Collino et al., in which they generated EVs from *DROSHA*-depleted MSCs, which were characterized by a general down-regulation of miRNAs without differing from those of naïve cells in quantity and surface marker expression [40]. Recent research has highlighted the therapeutic potential of miRNAs carried by sEVs, particularly in improving neonatal hypoxic-ischemic brain injuries [41,42,43,44,45]. MicroRNAs are selectively sorted into sEVs, and the miRNA cargo from sEVs differs from their source cells [46]. By performing a detailed analysis of the miRNA cargo using Next-Generation Sequencing (NGS) and Kyoto Encyclopedia of Genes and Genomes (KEGG) pathway analysis, our research group has recently revealed that, compared to their source cells, WJ-MSC-sEVs have a significantly higher abundance of specific miRNAs, including miR-21-3p and miR-186-5p [32]. Further highly enriched miRNAs in WJ-MSC-sEVs comprised miR-21-5p, miR-22-3p, miR-27b-3p and members of the let-7 family [32,39]. These miRNAs are suggested to target genes involved in WMI-related processes, including apoptosis, inflammation, and oligodendrocyte maturation [32]. Recently, we have demonstrated the functional role of miRNAs in WJ-MSC-sEVs in preventing and treating neuronal damage in premature WMI in vitro [39].

Based on these findings, sEVs and their miRNA cargo offer great potential for therapeutic use. The intranasal administration provides a promising delivery route for neurological injuries as it is minimally invasive and relatively easy to perform [22,23]. Nonetheless, more research is needed on the biodistribution of intranasally administered sEVs in vivo to understand which organs are targeted.

This study aims to investigate the biodistribution of WJ-MSC-sEVs and how miRNAs in WJ-MSC-sEVs impact neuroprotective and therapeutic outcomes in vivo in a rodent model of premature WMI.

## 2. Materials and Methods

### 2.1. Isolation of WJ-MSC-sEVs and DROSHA k.d sEVs

The research study received approval from the Ethics Committee of the Canton of Bern. To isolate small extracellular vesicles (sEVs) from human Wharton’s jelly-derived mesenchymal stromal cells (WJ-MSCs), we followed previously established protocols [39]. In brief, human umbilical cords were collected from healthy term deliveries (gestational age ≥ 37 weeks), with all donors having signed an informed consent. From the Wharton’s jelly of the umbilical cord, MSCs were isolated using enzymatic digestion, as described previously [47]. The cell culture medium was prepared as follows: Dulbecco’s modified Eagle’s medium (DMEM)/F12 (Thermo Fisher Scientific Inc., Waltham, MA, USA) was supplemented with 10% fetal bovine serum (FBS) (Thermo Fisher Scientific Inc.), 2 mmol/L GlutaMAX™ (Thermo Fisher Scientific Inc.), and 100 units/mL penicillin and 100 µg/mL streptomycin (Thermo Fisher Scientific Inc.).

For the isolation of naïve WJ-MSC-sEVs, the cells were cultured until passages 4 to 5, and when they reached 80% confluency, two washes with 1× phosphate-buffered saline (1× PBS) were performed. The medium was exchanged with FBS-free medium for sEVs isolation. The cell culture supernatant was collected after 36–40 hours (h), and sEVs were isolated through a two-step process. Initially, ultracentrifugation (UC) was performed in accordance with the protocol outlined by Théry et al. [48]. The UC was followed by size-exclusion chromatography (SEC), using an automatic fraction collector (IZON Science Ltd., Addington, Christchurch, New Zealand) in combination with Gen2 qEV single columns and 1× PBS as collection buffer. The SEC fractions’ protein and RNA concentrations were determined with a NanoVue Plus™ spectrophotometer (Biochrom, Holliston, MA, USA). The sEV-containing SEC fractions were combined, and their particle concentration was determined with a ZetaView^®^ x20 (Particle Metrix GmbH, Inning am Ammersee, Germany). The samples were stored at −80 °C until use. To isolate *DROSHA* k.d sEVs, WJ-MSC at passages 4 to 5 were transfected with a silencing RNA (siRNA) for *DROSHA* (Thermo Fisher Scientific Inc.), as previously described [39]. The isolation of sEVs was consistent with the description provided above. The experimental details were registered in the EV-TRACK knowledgebase (EV-TRACK ID: EV231018) [49].

### 2.2. Preparation of DiR-Labeled WJ-MSC-sEVs to Visualize Their Biodistribution

A DiR-dye (1,1′-Dioctadecyl-3,3,3′,3′-Tetramethylindotricarbocyanine Iodide, Thermo Fisher Scientific Inc., D12731) was used to label WJ-MSC-sEVs as follows: A DiR stock solution of 100 μM was added to the sEVs to a final concentration of 2.5 μM. The sEVs were then incubated on a shaker for 1.5 h at 37 °C. Thereafter, the DiR-labeled sEVs were ultracentrifuged at 100,000× *g* for 70 min to remove unbound dye. The pellet was resuspended in 110 μL of 1× PBS and immediately used for the intranasal application.

### 2.3. Animal Model of Premature WMI

**Figure 1:**
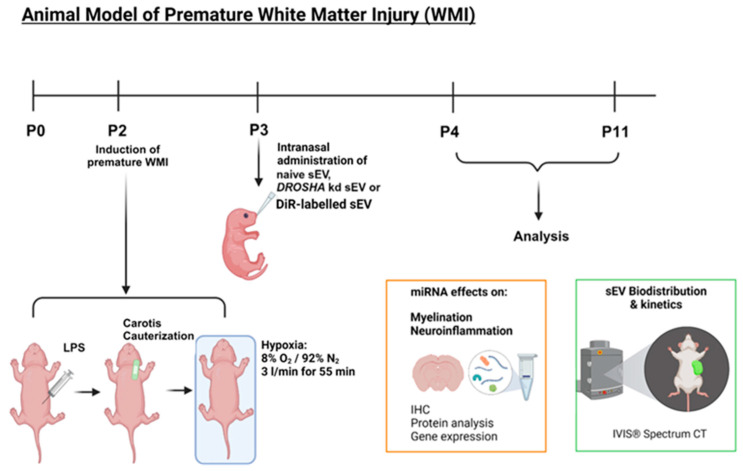
Animal model of premature white matter injury (WMI). A previously established rat model was used to analyze the biodistribution of DiR-labeled sEVs and the effect of naïve sEVs and *DROSHA* knockdown (k.d) sEVs. The rats were subjected to a multi-hit model of premature WMI at postnatal day (P) 2. In brief, 50 μg/μL LPS was administered intraperitoneally. Two hours (h) later, the left common carotid artery was cauterized, followed by 55 min of hypoxia (8% O_2_/92% N_2_ at a flow of 3 L/min) 2 h after the surgery. One day after the injury, at P3, 1 × 10^8^ particles per 10 g BW of either naïve sEVs, *DROSHA* k.d sEVs, or DiR-labeled sEVs were intranasally administered to the pups. The biodistribution (1 h, 6 h, and 24 h after administration) and the effect of the different sEVs (at P4 and P11) were subsequently analyzed. (sEVs: small extracellular vesicles; LPS: lipopolysaccharide; O_2_: oxygen; N_2_: nitrogen; IHC: immunohistochemistry. Created with BioRender.com).

To mimic premature WMI in rat pups, a multi-hit model with inflammatory and hypoxic-ischemic insults, previously established by our group, was used [29,30]. Approval for all animal procedures was obtained by the Veterinary Department of the Canton of Bern, Switzerland (reference number: BE113/2022; 35239). On postnatal day (P) 2, Wistar rat pups (Janvier Labs, Le Genest-Saint-Isle, France) were randomly divided into four experimental groups independent of their sex: mock-operated pups (control, n = 23), WMI + 1× PBS as vehicle (injured, n = 23), WMI + naïve sEVs (injured + sEVs, n = 24) or WMI + *DROSHA* k.d sEVs (injured + k.d sEVs, n = 23). To induce WMI, LPS (0.1 mg/kg in NaCl; *Escherichia coli* 0111:B4; Merck KGaA, Darmstadt, Germany) was intraperitoneally injected into all WMI groups 2 h prior to the cauterization of the left common carotid artery. After 2 h, the animals were subjected to hypoxia (8% O_2_/92% N_2_, 3 L/min) for 55 min (Figure 1), as previously described [30]. Twenty-four hours after the injury, the injured pups received an intranasal administration of 8 × 10^8^ particles per 10 g body weight (BW) of either naïve WJ-MSC-sEVs (injured + sEVs) or *DROSHA* k.d sEVs (injured + k.d sEVs), or the same volume of 1× PBS as vehicle (injured) (Figure 1). Per nostril, 100 units of hyaluronidase (H3884, Merck KGaA) in 1× PBS was administered intranasally 30 min before sEV or vehicle administration to ensure increased permeability of the nasal mucosa. For the healthy control animals, 0.9% NaCl was injected intraperitoneally; they were mock-operated, left at normoxic conditions, and did not receive any intranasal application.

The same rat model has been used to visualize the biodistribution of WJ-MSC-sEVs. The pups were randomly divided into three groups: mock-operated + DiR-labeled sEVs (n = 7), injured + DiR-labeled sEVs (n = 7), and injured + unlabeled sEVs (n = 4). Twenty-four hours after the injury, the pups received either 8 × 10^8^ DiR-labeled sEVs per 10 g BW or 8 × 10^8^ unlabeled sEVs per 10 g BW intranasally, depending on their respective group assignments (Figure 1).

Weight gain and natural mortality were documented for all pups throughout the experiment. The overall weight gain is represented in the Supplementary Figure S3.

### 2.4. Analysis of WJ-MSC-sEV Biodistribution In Vivo and Ex Vivo

To analyze the biodistribution of WJ-MSC-sEVs in vivo, the animals were imaged using an IVIS Spectrum CT (Perkin Elmer, Waltham, MA, USA). Fluorescent images were taken at emission/excitation wavelengths of 740/820 nm, either 1 h, 6 h, or 24 h after intranasal administration of DiR-labeled sEVs or unlabeled sEVs. To quantify the signal strength in the brain area, the total radiant efficiency of a region of interest (ROI) covering the head of each animal was measured at 1 h, 6 h, and 24 h after administration using the Living Image^®^ Software Version 4.8.0 (Perkin Elmer). The quantifications are displayed in Supplementary Figure S4. After 24 h, a computer tomography (CT) scan was also performed to create more detailed images of the biodistribution. To generate the CT images, a fluorescence imaging tomography (FLIT) image was produced by combining the fluorescent images taken at emission/excitation wavelengths of 740/820 nm with an X-ray image. Immediately after the FLIT imaging, the pups were euthanized with terminal sodium pentothal anesthesia (150 mg/kg BW), and the organs were dissected. Ex vivo fluorescent images of the lung, the liver, the spleen, and the gastrointestinal (GI) tract were taken.

### 2.5. Immunohistochemistry

The immunohistochemistry (IHC) analysis was performed as follows: The animals were sacrificed with terminal sodium pentothal anesthesia (150 mg/kg BW), and 4% para-formaldehyde (PFA) in 1× PBS was used for transcardial perfusion. The brains were first fixed in 4% PFA at 4 °C for 24 h and embedded in paraffin. Subsequently, 6 µm-thick coronal brain sections were cut from the paraffin-embedded tissue, then deparaffinized and rehydrated. Antigen retrieval was performed by heating the sections to 120 °C in 0.1 M sodium citrate buffer for 12 min. The sections were blocked at room temperature for 2 h with 1× PBS containing 0.05% Tween 20, 10% goat serum, and 1% BSA (blocking buffer).

To assess microglia accumulation based on the inflammatory insults, the animals were sacrificed at P4. The brain sections of the control (n = 6), injured (n = 7), injured + sEVs (n = 6), and injured + k.d sEVs (n = 6) pups were analyzed. After 1 h of blocking, the sections were incubated with an anti-ionized calcium-binding adaptor protein 1 (Iba1) antibody (ab5076, Abcam, Cambridge, UK), diluted 1:100 in blocking buffer, or an anti-CD68 antibody (ab31630, Abcam), diluted to 1:100 in blocking buffer overnight at 4 °C. The next day, the sections stained for Iba1 were treated with a peroxidase-coupled secondary antibody (Dako, Glostrup, Denmark) diluted to 1:200 in blocking buffer. Successful binding of the secondary antibody was visualized using 3,3′ diaminobenzidine (DAB) and the EnVision + System-HRP (Dako). Haematoxylin was applied to the sections as a counterstain to visualize cell nuclei. For CD68 staining, the sections were incubated for 1 h with an Alexa fluor^®^ 488-conjugated anti-rabbit antibody (a11008, Thermo Fisher Scientific) diluted to 1:200 in blocking buffer. Nuclear counterstaining was performed by incubating the sections with 4′,6-Diamidin-2-phenylindol (DAPI; Merck KGaA) for 5 min at room temperature.

The animals were sacrificed at P11 to assess mature OL quantification. Brain sections from the control (n = 6), injured (n = 4), injured + sEVs (n = 4), and injured + k.d sEVs (n = 5) were analyzed. The brain sections were blocked for 1 h at room temperature, as shown above. The brain sections were incubated with a rabbit anti-myelin basic protein (Mbp) antibody (ab40390, Abcam) diluted to 1:200 in blocking buffer at 4 °C overnight. The next day, a secondary Alexa fluor^®^ 488-conjugated anti-rabbit antibody (a11008, Thermo Fisher Scientific Inc.), diluted to 1:200 in blocking buffer, was applied on the sections for 1 h at room temperature prior to the nuclear counterstaining with DAPI (Merck KGaA) for 5 min at room temperature.

Images were acquired using a Leica DM6000 B microscope (Leica Microsystems, Wetzlar, Germany). Briefly, either brightfield images (Iba1) or fluorescent images (CD68 and Mbp) were taken. The percentages of Iba1-, CD68-, and Mbp-positive areas were subsequently measured using ImageJ (NIH, Bethesda, MD, USA). Positively stained cells in the white matter were characterized by distinct nuclear staining together with a DAB or Alexa fluor 488 signal, defined with the “Threshold” tool and applying the “Particles Analysis” plugin.

### 2.6. RNA and Protein Isolation

For RNA and protein isolation, rat pups at P4 or P11 were anesthetized with terminal sodium pentothal anesthesia (150 mg/kg BW). The rats were perfused with 1× PBS to flush out excess blood. The hemisphere ipsilateral to the cauterized common carotid artery of the brain was immediately frozen in liquid nitrogen and stored at −80 °C. Protein and RNA were extracted from the tissue using the QIAshredder and the All-prep DNA/RNA/Protein Mini Kit according to the manufacturer’s protocol (Qiagen, Hilden, Germany). The total protein concentration was determined using the Bicinchoninic Acid Protein Assay Kit (BCA1, Merck KGaA). The RNA concentration was measured with a NanoVue Plus™ spectrophotometer. The isolated RNA was considered pure and high-quality when the 260 nm/280 nm ratio was between 1.8 and 2.1.

### 2.7. cDNA Synthesis and Real-Time Quantitative Reverse Transcription Polymerase Chain Reaction (qRT-PCR)

Up to 3.5 μg of RNA was reverse-transcribed using the SuperScript IV First-Strand Synthesis System (Thermo Fisher Scientific Inc.). The synthesized cDNA was stored at −20 °C until use. The gene expression of Iba1, referred to as *Allograft Inflammatory Factor 1* (*Aif1* = Iba1), *Thousand And One Amino Acid Protein Kinase 1* (*Taok1*), *Tumor Necrosis Factor alpha* (*Tnfa*), *Caspase 3*, and *Bcl2-Associated Agonist Of Cell Death* (*Bad*) were quantified through the use of real-time qRT-PCR using the TaqMan primer and probes gene expression assays from Thermo Fisher Scientific Inc. for *Aif1* (ID: Rn00567906_g1), *Taok1* (ID: Mm00522816_m1) Tnfa (ID: Rn01525860_g1), *Caspase 3* (ID: Mm01195085_m1), and *Bad* (ID: Rn00575519_m1, all from Thermo Fisher Scientific Inc.). *Glyceraldehyde-3-Phosphate-Dehydrogenase* (*Gapdh*) was used as a housekeeping gene (Forward Primer: 5′-GCTCCTCCTGTTCGACAGTCA-3′, Reverse Primer: 5′-ACCTTCCCCATGGTGTCTGA-3′, and Probe: 5′-6FAM-CGTCGCCAGCCGAGCCACA-TAMRA-3′).

The PCR cycle programs were performed on a QuantStudio™ 7 Flex Real-Time PCR System (Thermo Fisher Scientific Inc.). In brief, 2 min at 50 °C and 20 s at 95 °C were followed by 45 cycles of 1 s at 95 °C and 20 s at 60 °C.

The gene expression was analyzed using the QuantStudio™ 7 Flex Real-Time PCR System Software. Data were expressed as the fold change relative to healthy, untreated rat brain RNA and normalized against *Gapdh* using the following formula: relative quantification (RQ) = 2^−ΔΔϹt^. The fold change was identified as downregulation if RQ < 1 and upregulation if RQ > 1.

### 2.8. Western Blot Analysis

Protein isolated from rat brains were separated through the use of sodiumdodecylsulphate–polyacrylamide gel electrophoresis (SDS-PAGE) on a 4–20% gradient gel (Bio-Rad, Hercules, CA, USA), transferred to PVDF membranes (IB401002, Thermo Fisher Scientific Inc.), and blocked for 1 h at room temperature with 5% non-fat dry milk dissolved in Tris-buffered saline containing 0.05% Tween 20 (WB-blocking buffer) (Merck KGaA). Protein expression was analyzed using the following antibodies diluted to 1:1000 in WB-blocking buffer: Iba-1 (ab178846, Abcam), Taok1 (ab197891, Abcam), and Mbp (ab40390, Abcam). Horseradish peroxidase-coupled donkey anti-rabbit antibody (NA9340, Merck KGaA) was used as a secondary antibody diluted to 1:1000 in WB-blocking buffer. Using the chemiluminescent Amersham ECL Prime Western Blotting Detection Reagent (Cytiva, Marlborough, MA, USA), the binding of the antibodies was detected on a C-DiGit Blot Scanner (LI-COR Biosciences, Lincoln, NE, USA).

### 2.9. Detection of Neuronal Cell Death: TdT-Mediated dUTP-Biotin Nick End Labelling (TUNEL) Staining and Analysis of Cleaved Caspase 3 and Bad

The brain sections of rat pups at P4 were analyzed for neuronal cell death using the TdT-mediated dUTP-biotin nick end labeling (TUNEL) method. Before the TUNEL staining, antigen retrieval was performed as described in Section 2.5. Next, the sections were washed three times, and 100 µL of the staining solution of the in situ cell death detection kit (Merck KGaA) was applied and incubated for 1 h at 37 °C.

To visualize neuronal cell death in the grey matter, the sections were incubated overnight at 4 °C with a primary antibody against neuronal nuclear antigen (NeuN) (rabbit anti-NeuN, ab177487, Abcam) diluted to 1:500 in 1× PBS containing 10% goat serum and 1% BSA (blocking buffer). The next day, the sections were incubated with an Alexa fluor^®^ 594-conjugated secondary antibody (A32740, Thermo Fisher Scientific Inc.) diluted to 1:200 in blocking buffer for 1 h at room temperature, followed by counterstaining with DAPI.

In addition, the sections were analyzed for cleaved Caspase 3 (9661, cell signaling) and Bad (32445, abcam) using IHC, as described in Section 2.5. The antibodies used were diluted 1:400 (cleaved Caspase 3) and 1:200 (Bad) in blocking buffer. Next to the protein analysis, the gene expression of *Caspase 3* and *Bad* was analyzed using qRT-PCR, as described in Section 2.7.

### 2.10. Statistical Analysis

Iba1, CD68, and Mbp expression between the four different groups (control, injured, injured + sEVs, or injured + k.d sEVs) were compared using one-way analysis of variance (ANOVA). The given *p*-values were adjusted for multiple comparisons according to Bonferroni. The differences between the groups were considered significant if adjusted *p*-values were lower than 0.05. Significance is indicated with asterisks (* *p* < 0.05, ** *p* < 0.01, *** *p* < 0.001, and **** *p* < 0.0001). All analyses were conducted with GraphPad Prism version 9.4.1 for Windows (Dotmatics, Boston, MA, USA).

## 3. Results

### 3.1. Intranasally Administered WJ-MSC-sEVs Target the Brain

**Figure 2:**
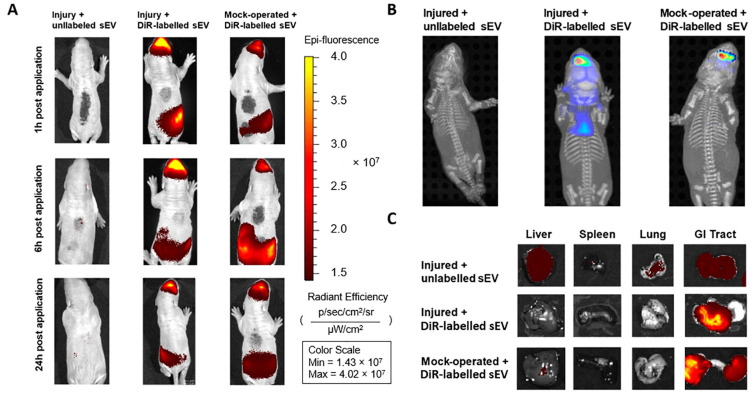
Biodistribution of intranasally administered DiR-labeled sEVs in vivo and ex vivo. (**A**) The DiR-signal was measured with a fluorescent scan 1 h, 6 h, and 24 h post-administration. Labeled sEVs were administered to either injured or mock-treated pups. The mock-treated pups were used as a reference to assess the biodistribution of intranasally applied sEVs under normal physiological conditions compared to the insult (injured). After 1 h, a fluorescent signal of the sEVs was observed in the brains of both groups that had received DiR-labeled sEVs. The signal remained in the brain until 24 h after administration. The group with non-labeled sEVs had no signal at any time point, indicating no auto-fluorescence from the sEVs. The grey spots on the backs of the animals served to identify them. (**B**) FLIT-imaging of the animals 24 h after sEV administration showed a strong signal in the brains of injured rat pups with DiR-labeled sEVs and some signal in control pups with DiR-labeled sEVs. No signal was observed in pups that had received unlabeled sEVs. (**C**) Ex vivo fluorescent imaging of the liver, spleen, lung, and gastrointestinal (GI) tract 24 h after sEV administration. The GI tract showed a high fluorescent signal in the groups that had received DiR-labeled sEVs. The other organs showed no signal in either group.

The in vivo model used is represented in Figure 1. The in vivo biodistribution of DiR-labeled WJ-MSC-sEVs after intranasal administration in rat pups at P3 was visualized using IVIS Spectrum CT. Control or injured animals with intranasal administration of DiR-labeled sEVs showed an accumulation of WJ-MSC-sEVs in the brain already one hour after administration (Figure 2A). The sEV signal could still be measured after 6 h and was above the detection level for at least 24 h in the brain. Animals that received non-labeled sEVs showed no signal at the three measured time points (Figure 2A). Evaluating the sEV biodistribution in the animals using FLIT-imaging proved the accumulation of DiR-labeled sEVs in the brains of the injured and the control group. The animals receiving non-labeled sEVs did not show any signal when subjected to FLIT imaging (Figure 2B), indicating no autofluorescence from the unlabeled WJ-MSC-sEVs.

Interestingly, a strong signal was detectable after 6 h in the middle abdominal area of the pups receiving the DiR-labeled sEVs (Figure 2A). To investigate the origin of the signal, ex vivo analyses of the GI tract, the liver, the spleen, and the lung were performed 24 h after the intranasal administration. The images confirmed an increased amount of DiR-labeled sEVs in the GI tract in both groups that had received DiR-labeled sEVs (Figure 2C). The liver, spleen, and lung did not show any strong signal in any of the groups.

### 3.2. Intranasal Administration of WJ-MSC-sEVs Ameliorates the Inflammatory Response in Experimental Premature WMI

The inflammatory response after WMI was analyzed in rat pups at P4 in terms of the expression of microglia markers Iba-1 and CD68 and by the expression of cytokines such as Tumor Necrosis Factor alpha (Tnfα) and Thousand And One Amino Acid Protein Kinase 1 (Taok1), a protein kinase known to be involved in inflammatory and apoptotic mechanisms that subsequently lead to adverse brain development [50,51,52], and is therefore involved in WMI-related processes.

**Figure 3:**
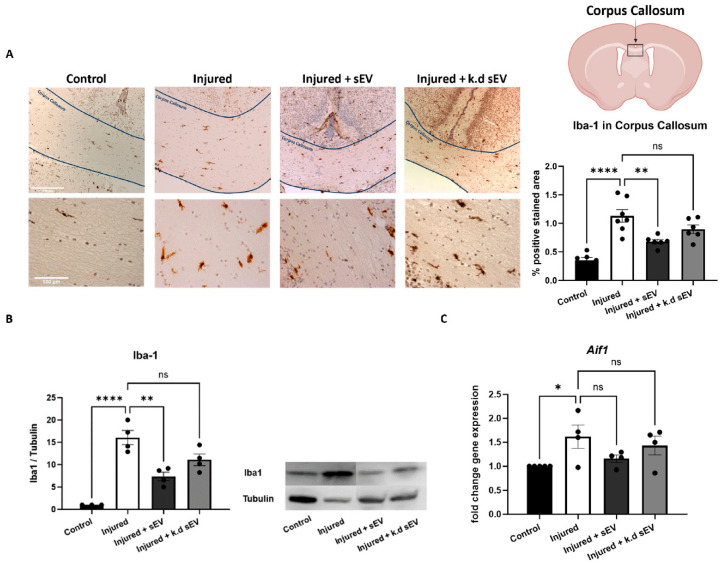
Analysis of Iba-1 mediated inflammation after induced WMI and sEV administration. Iba-1 expression was analyzed in rat pups at P4. (**A**) Iba-1 was significantly increased after injury compared to the control animals (control, n = 5). When naïve sEVs were intranasally administered in injured pups (injured + sEVs, n = 6), the Iba-1 expression was significantly reduced in the CC compared to the injured pups with vehicle administration (injured, n = 7). However, when *DROSHA* k.d sEVs were administered (injured + k.d sEVs, n = 6), Iba-1 expression did not significantly change compared to the injured group (scale bar upper row = 250 µm, scale bar lower row = 100 µm). (**B**) Western blot analysis revealed similar findings for the protein expression of Iba-1 (control: n = 3; injured: n = 4; injured + sEVs: n = 4; and injured + k.d sEVs: n = 4). (**C**) The same trend was observed for the gene expression of *Aif1* (allograft inflammatory factor 1 = Iba-1); however, there were no significant changes (control: n = 5; injured: n = 4; injured + sEVs: n = 4; injured + k.d sEVs: n = 4). (control: mock-operated; sEVs: small extracellular vesicles; k.d sEVs: *DROSHA* knockdown sEVs). Bars illustrate mean ± standard error of the mean (SEM), one-way ANOVA, **** *p* < 0.0001, ** *p* < 0.01, * *p* < 0.05, ns = not significant. Schematic of brain section created with BioRender.com).

The expression of Iba-1 was significantly increased in the corpus callosum (CC) of the injured pups compared to the mock-operated controls (*p* < 0.0001). The intranasal administration of naïve sEVs 24 h after the injury significantly decreased the amount of Iba1-positive cells in the CC compared to the pups of the injury group (*p* = 0.0025). On the other hand, when *DROSHA* k.d sEVs were administered, which have a reduced number of miRNAs, as we previously demonstrated [44], the amount of Iba1-positive cells in the CC did not decrease significantly in these pups compared to the injured pups that received vehicle only (*p* = 0.1766) (Figure 3A). Quantitative RT-PCR and Western blot analysis of Iba1 confirmed its reduced expression and transcription *(Aif1)* after naïve sEV or *DROSHA* k.d sEV administration (Figure 3B).

**Figure 4:**
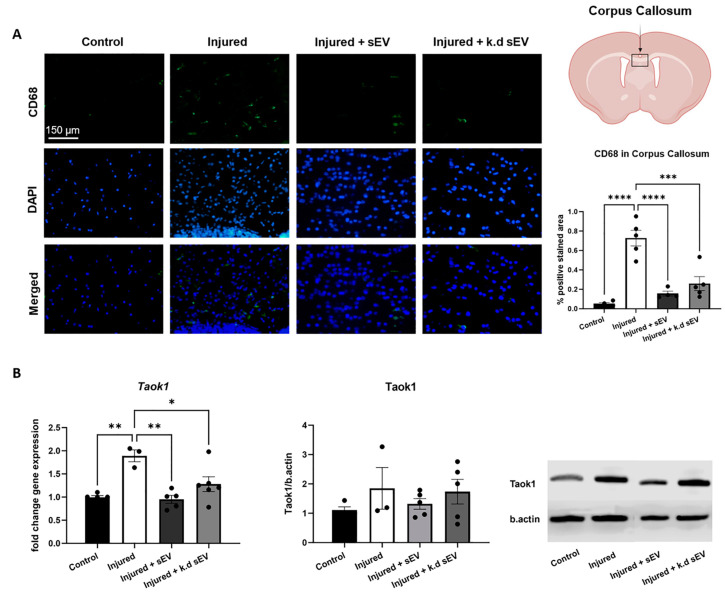
Analysis of CD68 and Taok1 expression after induced WMI and sEV administration. (**A**) In immunohistochemistry (IHC) analyses, CD68 expression in the corpus callosum (CC) increased significantly in the injured pups (n = 5) compared to the control pups (n = 4). The intranasal administration of naïve sEVs (injured + sEVs, n = 4) and *DROSHA* k.d sEVs (injured + k.d sEVs, n = 5) reduced the CD68 increase significantly compared to the injured pups with no administration of sEVs. (**B**) The gene expression of Taok1 in the brain of rat pups at P4 was compared between the control or injured pups with intranasal administration of either vehicle (injured, n = 3), naïve sEVs (injured + sEVs, n = 5), or *DROSHA* k.d sEVs (injured + k.d sEVs, n = 6). The injured pups receiving only vehicle significantly increased *Taok1* gene expression, while in both sEV groups (naïve sEVs or *DROSHA* k.d sEVs), it did not increase significantly compared to the control group (n = 5). The protein expression of Taok1 in the injured group and the injured + k.d sEV group was analyzed using Western blot. The protein expression shows an increase compared to the control or the injured + sEVs; however, the increase is not statistically significant (control: n = 4; injured: n = 3; injured + sEVs: n = 5; injured + k.d sEVs: n = 5). (Bars illustrate mean ± SEM, one-way ANOVA, **** *p* < 0.0001, *** *p* < 0.001, ** *p* < 0.01, * *p* < 0.05, ns = not significant, scale bar = 150 µm. Schematic of brain section created with BioRender.com).

The CD68 expression in the CC was significantly increased in the injured rat pups compared to the control pups (*p* < 0.0001). The intranasal administration of naïve sEVs 24 h after the injury significantly decreased CD68 expression relative to the injured pups (*p* < 0.0001). Interestingly, the intranasal administration of *DROSHA* k.d sEVs also led to a significant decrease in CD68 expression (*p* = 0.0003) (Figure 4A). In addition, the protein and gene expression of *Taok1* increased in injured pups compared to control pups. The intranasal administration of naïve sEVs, as well as *DROSHA* k.d sEVs, led to a decrease in *Taok1* transcription compared to the vehicle-treated injured pups. However, the protein expression did not change significantly (Figure 4B).

After injury, the gene expression of *Tnfa* increased significantly in the injured pups compared to control pups. Both naïve and *DROSHA* k.d. sEVs did not decrease the *Tnfa* gene expression compared to the injured group. Nonetheless, the intranasal administration of naïve sEVs showed a trend toward reducing the gene expression of *Tnfa* (*p* = 0.0933). In contrast, upon *DROSHA* k.d sEV administration, there was not even a hint of a reduction in the *Tnfa* expression (*p* = 0.9874) compared to the injured group, as presented in Supplementary Figure S1.

### 3.3. WJ-MSC-sEVs with Highly Abundant miRNA Cargo Increase OL Maturation

**Figure 5:**
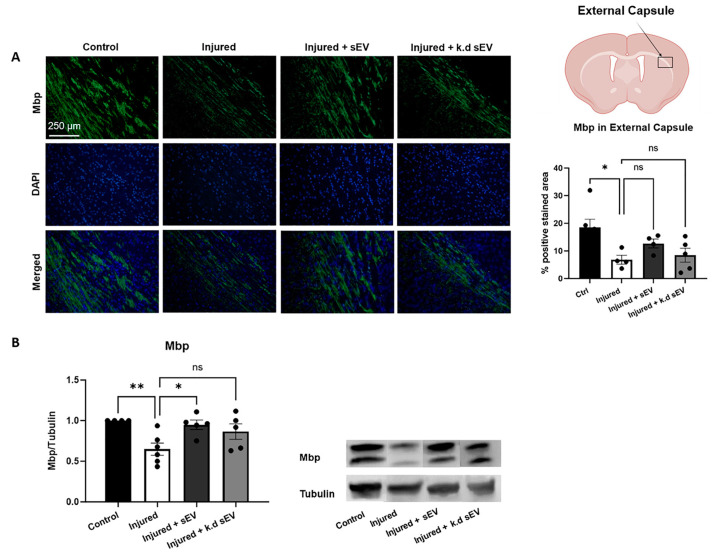
Analysis of oligodendrocyte maturation after WMI and sEV administration. (**A**) Immunohistochemistry (IHC) analysis of myelin basic protein (Mbp) in the external capsule of rat pups was performed at P11. Compared to control brains (n = 6), Mbp was significantly decreased in the injured group (n = 4). When the pups had received naïve sEVs (n = 4), the area positively stained for Mbp increased compared to the injured pups. When the pups had received *DROSHA* k.d sEVs (n = 5), the increase in the positively stained area was less pronounced. However, both sEV administrations did not significantly increase Mbp compared to the injured pups (injured + sEVs: *p* = 0.3845; injured + *DROSHA* k.d sEVs: *p* = 0.9628). (**B**) The protein expression showed a significant increase in Mbp when the rat pups had received naïve sEVs compared to untreated injured pups (*p* = 0.0190). When the rat pups had received *DROSHA* k.d sEVs, the expression did not increase significantly compared to injured pups (*p* = 0.0973) (control: n = 4; injured: n = 6; injured + sEVs: n = 5; injured + k.d sEVs: n = 5). (Bars illustrate mean ± SEM, one-way ANOVA, ** *p* < 0.01, * *p* < 0.05, ns = not significant, scale bar = 250 µm Schematic of brain section created with BioRender.com).

The expression of Mbp in the external capsule of rat pups at P11 was analyzed using IHC and Western blot. The percentage area of positively stained Mbp was significantly decreased after premature WMI injury in rat pups (*p* = 0.0085) compared to the control pups. When the pups received an intranasal administration of naïve sEVs, the area positively stained for Mbp increased compared to the injured ones. When the pups received *DROSHA* k.d sEVs, the increase in the positively stained area was less pronounced. However, both sEV administrations did not significantly increase Mbp compared to the injured pups (injured + sEVs: *p* = 0.3845; injured + *DROSHA* k.d sEVs: *p* = 0.9628). (Figure 5A). The protein expression of Mbp was significantly increased when the rat pups had received naïve sEVs compared to untreated injured pups (*p* = 0.0190). However, when the rat pups had received *DROSHA* k.d sEVs, the expression did not increase significantly compared to injured pups (*p* = 0.0973) (Figure 5B).

### 3.4. Hippocampal Cell Death Is Not Increased in Experimental Premature WMI

Neuronal cell death was validated by the amount of DNA strand breaks with fluorescent TUNEL-staining as well as the IHC of cleaved Caspase 3 and Bad. All of the staining was double-stained with the neuronal nuclear antigen marker (NeuN) in the cornu ammonis (CA1) area of the hippocampus. Nuclear counterstaining was performed using DAPI. Brain sections were analyzed at P4 24 h after intranasal administration of either vehicle, naïve sEVs, or *DROSHA* k.d sEVs. TUNEL+ cells correlating with NeuN expression (TUNEL+NeuN+) were detected only occasionally in the hippocampal region in all analyzed groups. No significant differences in cleaved Caspase 3 or Bad were detected in the hippocampal region between all of the analyzed groups, both at the gene and protein expression level. The findings indicate no elevated neuronal cell death in this rodent model of premature WMI, as presented in Supplementary Figure S2.

## 4. Discussion

In this study, we provide evidence that intranasally administered sEVs target the brain after WMI and remain at the injury site for at least 24 h. We further confirmed that miRNAs carried by WJ-MSC-sEVs are involved in the observed neuroprotective effects of WJ-MSC-sEVs in the experimental premature WMI in an in vivo model. Mature miRNAs are considerably involved in reducing Iba1-expressing activated microglia and preventing Mbp-loss after WMI.

To successfully use sEVs in clinical applications, it is essential to understand their biodistribution after administration. The delivery method is crucial to the efficacy of therapeutic substances. Intranasal administration offers a promising delivery route for neurological disorders as it targets the brain more specifically than other delivery methods. Intranasal administration is also minimally invasive and relatively easy to apply, and thus, it is well-suited for the treatment of neonatal disorders [22,23,53].

In this study, we present that intranasally administered WJ-MSC-sEVs reach the brain, where they remain present for at least 24 h after administration. Interestingly, in vivo fluorescent and FLIT images show a higher uptake of sEVs into the brains of rat pups subjected to WMI than into those of control pups. Thus, injury might facilitate the uptake of vesicles into the brain. These findings align with other studies, suggesting the enhanced uptake of particles after inflammation due to the leakage of the blood–brain barrier [54,55]. Consistent with previous research, including our own, we could not detect any signal of WJ-MSC-sEVs in the liver or spleen after intranasal administration [30]. Compared to other administration methods, such as intravenous delivery, the uptake and the potential side effects on these organs are reduced upon intranasal application [56,57]. We could not detect any signal in the lungs, although intranasal administration is likely to interfere with the respiratory pathways, as they cover most of the nasal surface area [22]. In addition, our research group has recently found a small portion of sEVs in the trachea 30 min and 3 h after administration [30]. However, these sEVs were applied during the injury and not 24 h after, as in this study. In addition, they were labeled with a different dye (IRDye^®^ 800CW), possibly explaining the differences from the current study. The findings of the present study correlate with a study by Liu et al., who did not observe pulmonary effects after intranasal administration of MSC in a preclinical neonatal lung disease model [58]. We further observed a strong sEV signal in the GI tract, which is unsurprising, as intranasally administered drugs are generally cleared via mucociliary clearance from the nasal cavity into the GI tract [59]. Nevertheless, using FLIT imaging, we detected a robust fluorescent signal in the brains of injured pups 24 h after administration. Based on these findings, our results prove that the intranasal application of WJ-MCS-sEVs targets the brain, where they remain at the site of injury for at least one day.

Consistent with our previous results, the rodent model of premature WMI used in this study showed microglia activation in the corpus callosum (CC), constituting the brain’s most important region of white matter [29]. Premature birth is often accompanied by inflammatory and hypoxic-ischemic insults leading to WMI [3,10]. Specifically, these insults lead to an increase in ROS, which, in turn, activates microglia and polarises them towards the pro-inflammatory M1 phenotype [2,10]. M1 microglia lead to the arrest of OL maturation and, thus, to the adverse outcomes of WMI [12,13,14,15]. We and others have shown an anti-inflammatory effect of MSC-sEVs in premature brain injuries [27,29,43,44,60,61].

In line with our findings, which suggest that miRNAs play a crucial role in the observed anti-inflammatory effects of MSC-sEVs, Xin et al. discovered that miR-21-5p carried by sEVs, enhanced polarisation towards the anti-inflammatory M2 microglia phenotype in neonatal hypoxia-ischemia [43,44]. In accordance with these studies, we have previously shown that miR-21-5p, which is involved in preventing neuronal apoptosis and promoting OL maturation, is highly abundant in WJ-MSC-sEVs [32,39]. Additionally, Chu et al. preconditioned MSCs with hydrogen sulphide, resulting in the upregulation of miR-7b-5p in the released EV, subsequently improving cognitive impairments following hypoxia-ischemia in neonatal mice [61].

During premature WMI, both feto–maternal inflammation and neonatal hypoxia/ischemia cause the activation of brain-resident immune cells, such as microglia [12]. This study demonstrates that only naïve sEVs with a marked abundance of mature miRNAs could decrease Iba1-positive cells in the CC and thus reduce activated microglia. However, *DROSHA* k.d sEVs, containing less of the mature miRNAs, did not significantly decrease the number of Iba1-positive cells. Interestingly, the administration of both types of sEVs, naïve and *DROSHA* k.d sEVs, reduced CD68-positive cells. CD68 is more specifically involved in phagocytosis, whereas Iba1 indicates activated microglia but is not limited to phagocytic activity [62].

Phagocytosis is an essential component of the immune response, involving the engulfing and digestion of cellular debris and pathogens. Microglial phagocytosis depends on multiple receptor mechanisms, which are, in general, Toll-like receptors (TLRs) that can bind pathogens and the triggering receptor expressed on myeloid cells 2 (TREM-2) that can detect apoptotic cellular debris [63]. The interaction of sEVs with receptors such as TLRs or TREM-2 is complex and not yet completely understood [64,65]. They may interact with these receptors and regulate their activity via cargo other than miRNAs, such as proteins [64,65]. In Alzheimer’s disease, sEVs containing abundant amyloid-beta (Aβ) peptides can activate the TLR signaling pathways to decrease the symptoms of AD at the early stage [64]. Moreover, in Parkinson’s disease, sEVs can transfer alpha-synuclein to the brain. This transfer increases the expression of several TLRs, ultimately leading to an inflammatory reaction [64]. Additionally, sEVs themselves contain membrane receptors, such as TREM-2 or TLRs. These integrated receptors can be transferred to immune cells, enhancing the phagocytic response to inflammatory insults [65,66]. Thus, *DROSHA* k.d sEVs might still present these receptors in their membrane, thereby decreasing CD68 in induced WMI. Nevertheless, we did not analyze the expression of these receptors in our samples. Of note, it should further be considered that sEVs could be phagocytosed [67]. However, the engulfment of sEVs would instead result in an increase in CD68. This increase would indicate an enhancement of harmful activated microglia instead of neuroprotection after sEV administration. The phagocytosis of sEVs is a very complex process, and the exact mechanism of how the sEVs interact with activated microglia requires further investigation.

Additionally, the maturation of a subset of miRNAs can follow a *DROSHA*-independent pathway [68]. Hence, in our experimental model, it is plausible that specific miRNAs could persist within the *DROSHA* k.d sEVs. Considering this information and our findings, it becomes evident that the complete interaction mechanism of sEVs with phagocytic events does not solely rely on the presence of *DROSHA*-dependent mature miRNAs. However, further investigation is required to fully understand this mechanism.

Our results further demonstrate the significance of the miRNA cargo of sEVs in its capacity to promote OL maturation. Immature OLs do not express Mbp, whereas mature, myelinating OLs express and secrete Mbp [69]. Therefore, Mbp can serve as a marker for analyzing the degree of myelination. We and others have previously shown that MSC-sEVs can improve Mbp expression in premature neonates and thus ameliorate OL maturation [30,60,70]. This study shows a significant decrease in Mbp expression following induced WMI in injured pups compared to the control pups. However, based on Western blot analysis, the intranasal administration of naïve sEVs following WMI increased the Mbp expression significantly compared to injured pups receiving only vehicle. This was not the case when the pups received *DROSHA* k.d sEVs. Consistent with our discovery, a recent study by Huo et al. demonstrated that the transfer of miR-128-3p from sEVs isolated from neuronal stem cells significantly increases Mbp in OPCs [70]. Thus, we suggest that mature miRNAs in sEVs are crucial for promoting OL maturation after premature WMI.

The role of Taok1 in neurological disorders is multifaceted and has been associated with WMI-related processes, such as inflammation and apoptosis [50,51,52]. Our research group has previously shown in vitro that reducing Taok1 expression upon sEV administration is significantly dependent on the sEV miRNA cargo [39]. However, the in vivo data presented in this work show a tendency to decrease Taok1 expression not only after the administration of naïve sEVs but also with *DROSHA* k.d sEVs. Thus, in vivo, the ability of sEV to regulate protein kinases, such as Taok1, appears to not only rely on their miRNA content. Given the diverse roles of Taok1 in a living system, such as its ability to regulate microtubule dynamics and cell division, and its involvement in various signaling pathways, it is not surprising that the mechanisms controlling Taok1 expression in vivo are more complex [51]. Next to miRNAs, Taok1 might be regulated in vivo by other sEV cargoes, including proteins and peptides, which could interact with Taok1 in various ways. Taok1 may form complexes with proteins delivered by sEVs, potentially influencing its substrate specificity and phosphorylation of downstream targets [71]. In addition, specific peptides carried by sEVs may compete with docking sites or cell-targeting proteins of Taok1, leading to its inhibition [72]. Considering the different processes that control protein kinases in vivo is crucial for a comprehensive understanding of the underlying mechanisms of how sEVs affect the regulation of Taok1. It is worth mentioning that the gene expression of the pro-inflammatory cytokine *Tnfa* was significantly increased in experimental premature WMI at P4. The intranasal administration of naïve sEVs appeared to have a stronger tendency to decrease the *Tnfa* expression compared to *DROSHA* k.d sEVs (*p* = 0.0933 compared to *p* = 0.9874, respectively). We have already previously shown that the intranasal administration of WJ-MSC-sEVs reduced *Tnfa* in experimental premature WMI [29]. The sEVs were then administered during the injury, in contrast to the present study, in which the administration was performed 24 h after the damage induction. Therefore, the effect in the present study could be less pronounced because of the timing of the administration. Nonetheless, the effects of *DROSHA* k.d tend to have a lower inhibitory effect compared to naïve sEVs, indicating the relevance of miRNA in sEVs to reduce pro-inflammatory cytokine expression. This aligns with previous research, identifying the miRNA cargo of sEVs as the main regulator of inflammatory cytokines upon neonatal brain injuries [73,74,75]. For instance, Zhang et al. recently discovered that miR-18a-5p derived from MSC-EVs ameliorated inflammatory response and oxidative stress following subarachnoid hemorrhage, associated with a downregulation of pro-inflammatory cytokines, including *Tnfa* [75].

In contrast to prior studies indicating the presence of grey matter cell death in PMB [16,17,18], we did not observe cell death in the hippocampal area of rats at P4 after induced WMI. This could be in part because grey matter damage is mainly related to the more severe cPVL [76,77,78,79], while several studies of diffuse WMI report the absence of neuronal apoptosis [80,81,82]. It is worth mentioning that the TUNEL assay used might have been an inadequate choice, leading to false-negative results as it primarily detects late-stage apoptosis, while an analysis of early apoptotic events, characterized by other cellular changes, might have been more appropriate. Alternative analytical methods, like measuring annexin V expression, mitochondrial membrane potential, or caspase 3 expression, could better detect early apoptosis [83]. Therefore, in addition to the TUNEL assay, we analyzed the protein and gene expression of Caspase 3 and Bad, both known apoptosis markers. However, this analysis did not detect neuronal apoptosis in any of the groups. Premature WMI typically involves damage to white matter regions, whereas grey matter is relatively spared in most cases [18]. In diffuse WMI, the lack of myelinating OLs predominates over grey matter apoptosis [80,81,82]. It appears that neuronal apoptosis is not present in the grey matter areas, but rather, the failure of proper myelination of neurons leads to the phenotype of diffuse WMI [81,82]. These findings highlight the complexity of the relationship between grey matter and PMB. The involvement of grey matter cell death in premature WMI is a topic of ongoing research and debate. Additional investigations, including studies with a specific focus on the timing of the analysis, are needed to completely understand neuronal apoptosis in the context of WMI.

## 5. Conclusions

In conclusion, our results highlight the central role of miRNAs carried by WJ-MSC-sEVs in exerting neuroprotective effects of premature WMI. In particular, mature miRNAs emerge as essential factors exerting a dual effect by attenuating Iba1-marked microglia activation and promoting OL maturation after premature WMI. This study further presents convincing evidence regarding the intranasal administration of sEVs targeting the brain after WMI and their sustained presence at the injury site for at least 24 h. This suggests that miRNAs could potentially be used to mitigate inflammatory responses and promote myelination in impaired neurodevelopmental conditions.

However, as Drosha has key functions in miRNA-independent transcriptional regulation, RNA processing, and genome integrity maintenance [84], the reduced levels of Drosha following *DROSHA* k.d could have a wide-ranging impact on the overall gene expression. In addition to the reduced miRNA content in these vesicles, this possibly contributes to the differential effects observed with *DROSHA* k.d sEVs, which have to be addressed in future experiments. Nevertheless, the findings of this study open avenues for therapeutic interventions involving miRNAs carried by WJ-MSC-sEVs to address neuroprotective needs in cases of premature WMI. The intranasal administration of sEVs is highlighted as a very promising method for delivering these therapeutic agents to the brain.

## Data Availability

The datasets generated and/or analyzed during the current study can be obtained from the corresponding author upon reasonable request.

## Supplementary Materials

The following supporting information can be downloaded at: https://www.mdpi.com/article/10.3390/cells13060543/s1, Figure S1: *Tnfα* gene expression after WMI, Figure S2: Analysis of grey matter cell death after WMI, Figure S3: Overall weight gain of the rat pups, and Figure S4: Quantitative data of the biodistribution of WJ-MSC-sEVs.
