# Supplementary material for: All but Small: miRNAs from Wharton’s Jelly-Mesenchymal Stromal Cell Small Extracellular Vesicles Rescue Premature White Matter Injury after Intranasal Administration
Source: Cells. 2024 Mar 19;13(6):543. doi: 10.3390/cells13060543 (PMC10969443; doi:10.3390/cells13060543)
Supplement: Supplementary file 1 [file cells-13-00543-s001.zip › cells-2813517-supplementary.pdf]

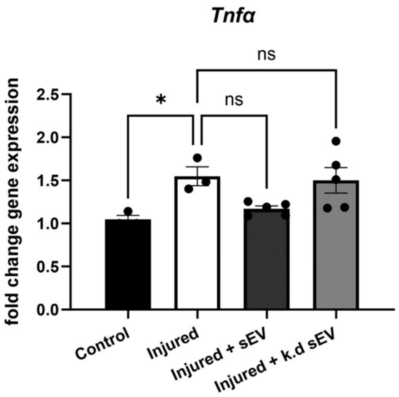

Figure S1: *Tnfa* gene expression after WMI.
The gene expression of *Tnfa* in the brain of rat pups at P4 was compared between control pups and injured pups having received an intranasal administration of either vehicle (Injured), naïve sEV (Injured + sEV), or *DROSHA* k.d sEV (Injured + k.d sEV). The injured pups receiving only vehicle showed a significant increase of *Tnfa*. However, in both sEV-treated groups (naïve sEV, or *DROSHA* k.d sEV), the *Tnfa* gene expression did not decrease significantly compared to the injured group (Bars illustrate mean ± SEM).

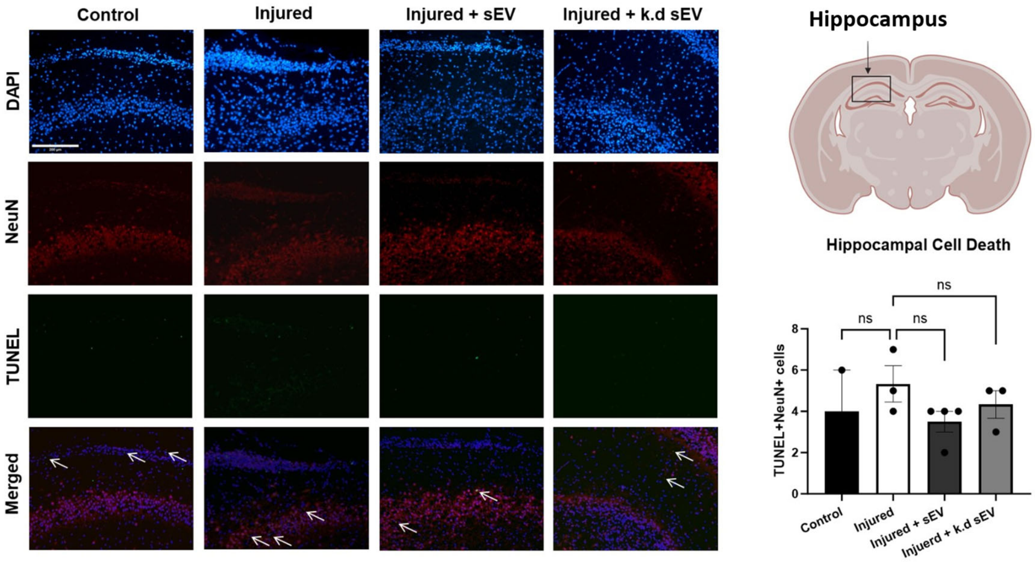

Figure S2: Analysis of grey matter cell death after WMI.
Neuronal cell death was analysed using fluorescent TUNEL-staining, co-stained with the neuronal nuclear antigen marker (NeuN). Nuclear counter-staining was performed with DAPI. TUNEL+NeuN+ cells were detected only occasionally for each group in the hippocampal region CA1, as indicated by the white arrows in the merged images. No significant difference was observed between all analysed groups. (Bars illustrate mean ± SEM, one-way ANOVA, ns = not significant, scale bar = 150 μm. Schematic of brain section created with BioRender.com).

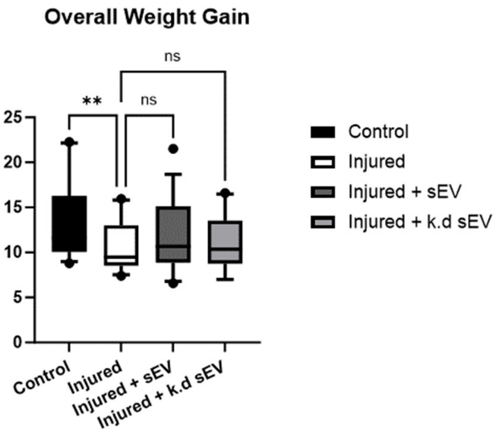

Figure S3: Overall weight gain of the rat pups.
The overall weight gain of the rat pups during the experiment has been documented. Injured pups had a significantly lower weight gain compared to the control group. However, neither the intranasal administration of naïve sEV ($p = 0.4985$) nor *DROSHA* k.d sEV ($p = 0.8860$) did change the weight gain significantly compared to the injured group, possibly due to high variability in litter size and the dams' behaviour during the experiment (Bars illustrate mean ± SEM, one-way ANOVA, ns = not significant).

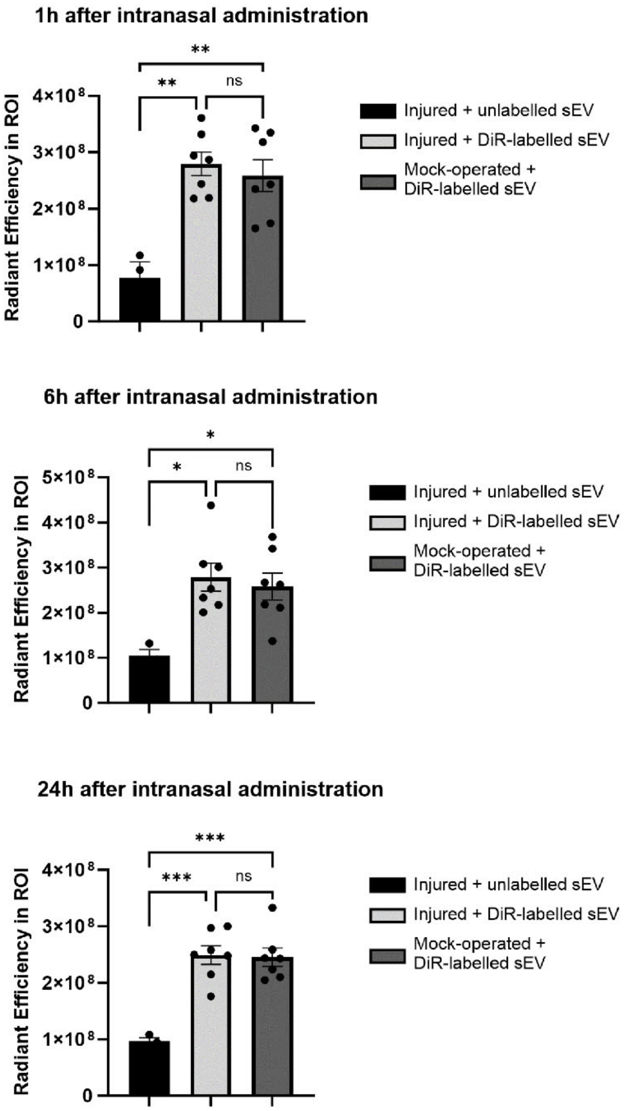

Figure S4: Biodistributionof intranasally administered WJ-MSC-Sev

The total radiant efficiency ((p/sec/cm2/sr)/(µW/cm2)) of a defined region of interest (ROI) covering the head of each animal has been measured at 1 h, 6 h and 24 h after administration using the Living Image Software (Perkin Elmer). The animals receiving DiR-Iablelled sEV had a significantly higher radiant efficiency. However, no significant difference could be measured between injured or mockoperated animals (Injured + unlabelled sEV: n = 3, Injured + DiR-labelled sEV: n = 7, Mock-operated + DiR-labelled sEV: n = 7). (Bars illustrate mean ± SEM, one-way ANOVA, ***$p < 0.001$,**$p < 0.01$,*$p < 0.05$, ns = not significant.).
